# Total monomeric anthocyanin concentration estimation of red wines based on color data using multivariate statistical analysis and machine learning

**DOI:** 10.1002/jsfa.70815

**Published:** 2026-06-16

**Authors:** Emirhan Atlayan, Berkay Berk, Sevcan Unluturk

**Affiliations:** ^1^ Department of Food Engineering, Faculty of Engineering Izmir Institute of Technology Izmir Turkey

**Keywords:** image processing, machine learning, multivariate statistics, total monomeric anthocyanin, wine

## Abstract

**BACKGROUND:**

Red wine is a high‐value grape derivative containing complex chemical compounds that dictate its quality. Qualitative and quantitative determination of these compounds is essential for standardizing and classifying the product. This proof‐of‐concept study integrates chemometric methods and machine learning algorithms to establish predictive models for estimating total monomeric anthocyanin (TMA) concentration in red wines using non‐destructive colorimetric data.

**RESULTS:**

Images were generated and analyzed using wine samples at three liquid depths (2, 3 and 4 mm) across three color spaces (RGB, HSV and Lab). To predict TMA content from extracted digital image features, partial least squares regression (PLSR), random forest (RF) and artificial neural network (ANN) models were evaluated using external validation and stratified five‐fold cross‐validation. Optimal imaging configurations were identified as 2 mm + HSV for PLSR and 2 mm + Lab for both RF and ANN. Cross‐validated performance metrics yielded mean test *R* ^2^ values of 0.897 ± 0.019 for PLSR, 0.960 ± 0.011 for RF and 0.973 ± 0.020 for ANN. Corresponding root mean square errors were 0.252 ± 0.032, 0.174 ± 0.023 and 0.113 ± 0.037, respectively. A one‐way analysis of variance confirmed significant differences among the models (*P* < 0.05), and Tukey's honestly significant difference grouped the non‐linear RF and ANN models together, separating them from PLSR.

**CONCLUSION:**

Under these controlled experimental conditions, non‐linear methods (RF and ANN) demonstrated significantly stronger predictive performance than the linear PLSR model (*P* < 0.05). This methodology offers a rapid, non‐destructive and highly promising approach for the accurate determination of TMA. © 2026 The Author(s). *Journal of the Science of Food and Agriculture* published by John Wiley & Sons Ltd on behalf of Society of Chemical Industry.

## INTRODUCTION

Red wine has a complex chemical matrix consisting of hundreds of compounds at different concentrations including ethanol, sugars, organic acids and various phenolics.^1^ One of the most important aspects of wines is their color, with the redness of wines mainly being caused by phenolic color pigments called anthocyanins.^2^

During winemaking, several complex chemical reactions take place, such as co‐pigmentation and polymerization, and these compounds are converted into multiple derivatives of polymeric anthocyanins that strongly affect the stability and variety of different colors and hue in red wine.^3^ During fermentation and primarily in maturation, the content of monomeric anthocyanins continuously decreases. During winemaking, the concentration of monomeric anthocyanins decreases, but the new products derived from anthocyanins result in a stable red color in wine.^4^

The implementation of spectrophotometric techniques in combination with chemometrics is one of the most effective methods in quantification of chemical species across various matrices. The qualification and quantification of chemical composition of wines are detected via several techniques including UV‐visible spectroscopy,^5^ NMR spectroscopy,^6^ electronic tongue^7^ and Fourier transform mid‐infrared spectroscopy.^8^ However, these techniques require specialized instrumental knowledge and are generally costly and destructive. Recently, the development of computer‐assisted, low‐cost, non‐destructive digital image processing methods has been gaining attention.

Leveraging modern computing power, it is possible to turn a webcam and computer into an optical detector that can quantify chemical compounds precisely using multivariate statistics.^9^ The digital images captured by a webcam consist of pixels (i.e. picture elements), each represented by an RGB triplet (R: red; G: green; B: blue) with varying intensities that define its color. It is possible to convert this color space into other formats, such as HSV (hue, saturation, value) and CIE‐Lab (lightness, *a*: redness to greenness; *b*: blueness to yellowness). These color spaces separate luma, which refers to image intensity, from chroma, which pertains to color information. Various chemometric methods, such as principal component analysis (PCA), partial least squares regression (PLSR) and partial least squares discriminant analysis (PLS‐DA), are commonly used to analyze the multivariate color data extracted from digital images of diverse food samples.^10^ For example, Herrero‐Latorre *et al*.^11^ demonstrated that multivariate statistical analysis and chemometrics applied to RGB digital images can be used to detect and quantify adulteration in aged wines. In addition to linear regression models like PLSR, there are tree‐based regression models that can capture non‐linear relationships between data, such as random forest (RF) regression. In recent years, artificial neural networks (ANNs) have emerged as powerful machine learning tools, capable of capturing complex non‐linear relationships among variables.^12^ Unlike models such as PLSR or RF, artificial neural networks (ANNs) can automatically learn complex non‐linear feature interactions without the need for explicit feature engineering. This capability can be further enhanced when combined with dimensionality reduction or regularization techniques such as L1 regularization (LASSO). Mat Nawi *et al*.^13^ used ANN on visible and shortwave near infrared spectra data to classify various sugarcanes based on their total soluble solid (°Bx) content. Kondo *et al*.^14^ used ANN and color features extracted from machine vision system to predict acid and sugar content of *Iyokan* orange fruit. Omid *et al*.^15^ used ANN to develop an intelligent sorting system for pistachio nut varieties.

To the best of our knowledge, no previous study has explored predicting anthocyanin concentration in red wines using RF or artificial neural network regression based on features extracted from digital image analysis. The present study develops and compares multivariate statistical and machine learning regression models (PLSR, ANN and RF) to predict total monomeric anthocyanin (TMA) concentration in red wines from digital image data. For this purpose, an analytical device for image acquisition and analysis was developed to monitor TMA levels.

## MATERIALS AND METHODS

### Red wine samples

In total, 43 bottles of red wine samples were purchased from local markets in the city of İzmir (Turkey). The samples consisted of both single‐varietal and blended wines produced from Syrah, Boğazkere, Öküzgözü, Çalkarası, Cabernet Sauvignon and Merlot grape varieties. Wines from multiple vintages (2022–2024) with varying alcohol contents, soluble solid contents and anthocyanin concentrations were intentionally selected to maximize compositional variability within the dataset. The use of commercially available wines enabled inclusion of naturally occurring variations associated with grape variety, vintage, processing conditions and maturation history. No treatment was applied before measurement. To avoid oxidation, the samples were analyzed immediately after being opened.

### Construction of the imaging system

**Figure 1:**
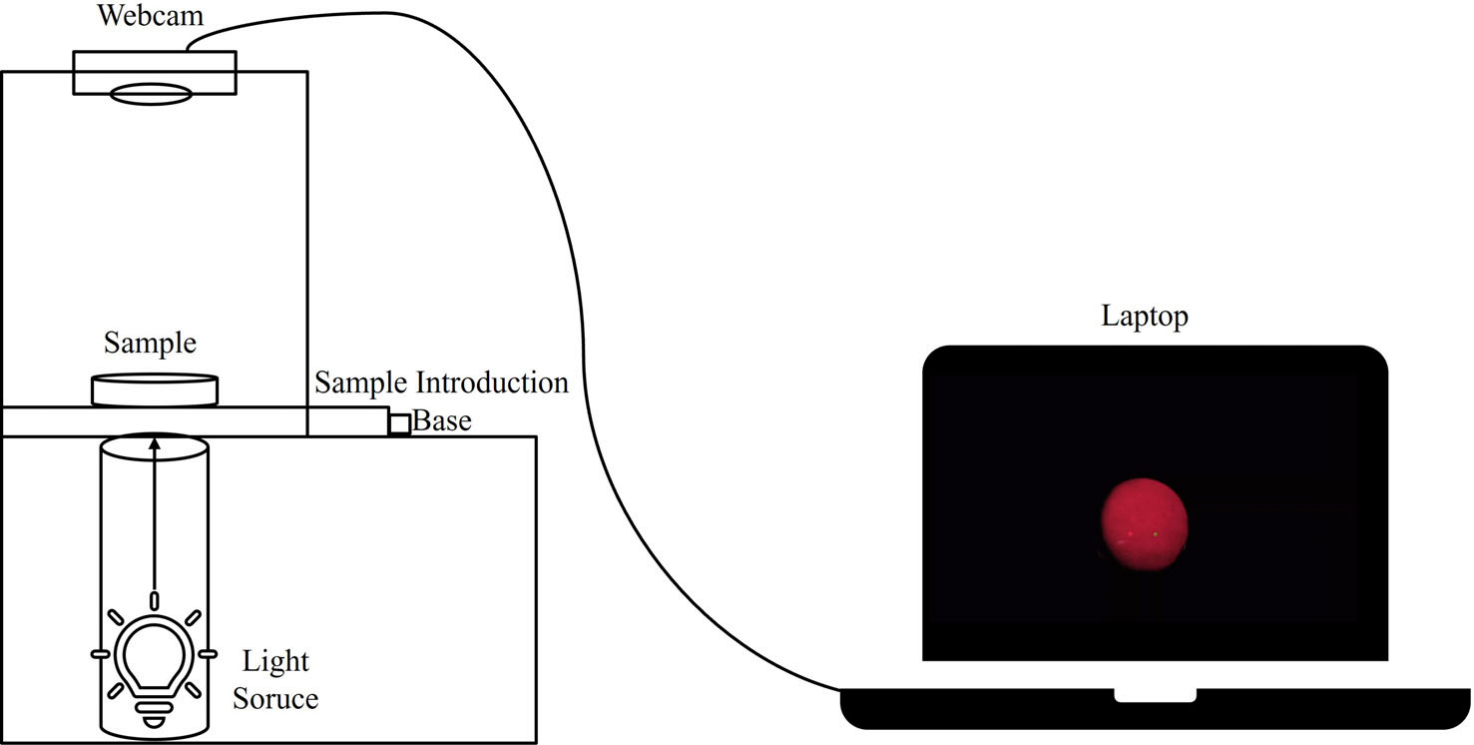
Representative scheme of photobooth cabinet.

A custom‐built photobooth cabinet was developed to capture digital images of wine samples under standardized and reproducible conditions (Fig. 1). The cabinet consists of two compartments. The lower compartment contains the LED light source, which emits a stable and constant illumination of 18 lux, measured at the sample position. The light passes vertically through a defined light path to ensure uniform exposure across the entire sample area. The upper compartment contains the imaging system. A standard webcam camera (SC‐HD02; EVEREST, Istanbul, Turkey) is mounted directly above the sample stage to capture the transmitted light. The stage itself is constructed as a sliding rail system, allowing Petri dishes containing wine samples to be positioned precisely above the illumination source. This arrangement ensures consistent alignment of the sample with the optical axis of both the light source and camera. The entire cabinet is enclosed in a light‐tight structure, preventing interference from ambient light and ensuring stable imaging conditions. This controlled environment guarantees that differences in recorded images originate from the sample itself, rather than fluctuations in illumination or external factors.

The design is based on transmitted light imaging, in which light passes through the wine sample before being detected by the camera. This approach was preferred over reflected light because by capturing the interaction of light with the full depth of the liquid, transmitted imaging provides more robust and reproducible information on color intensity and clarity, both of which are critical parameters in wine characterization.

### Automated algorithm for capturing digital images

**Figure 2:**
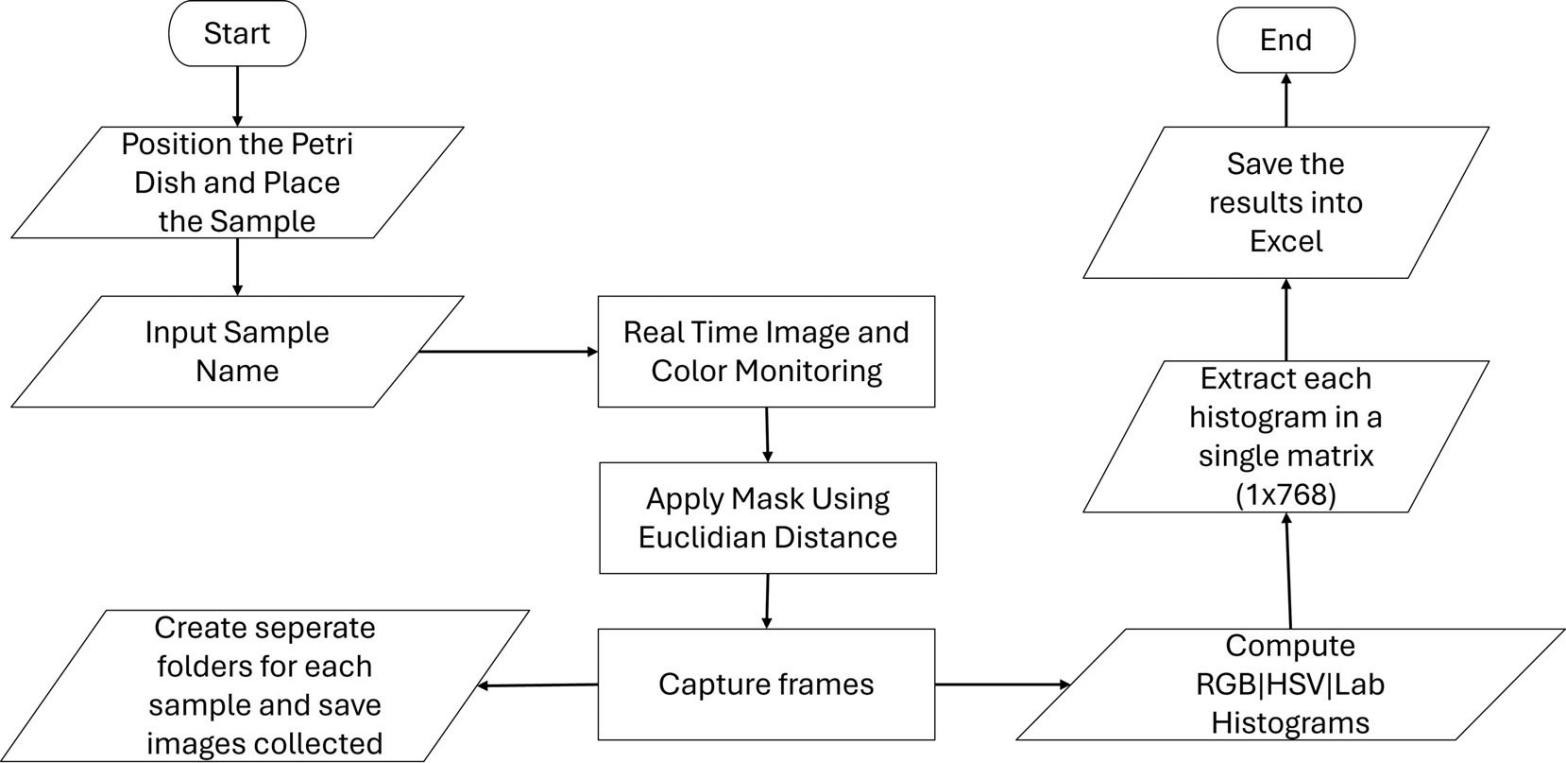
Block diagram of the algorithm used in this study to acquire digital images and extract color features.

A Python script (https://www.python.org) was developed to capture and process color data from wine samples using a webcam and a graphical user interface (GUI). The script initializes essential libraries (OpenCV, NumPy and Tkinter) and applies a circular mask to isolate the region of interest (ROI) using the Euclidean distance formula. It captures consecutive frames, converts them to RGB, HSV and Lab color spaces, extracts ROI histograms, flattens them into a 1 × 768 vector, and saves both images and numerical data. The live webcam feed continuously updates, displaying real‐time mean RGB, HSV and Lab values as overlays and visually marking the ROI. A Tkinter‐based GUI enables users to input sample labels and capture data with a single click. The algorithm used in this study is demonstrated in Fig. 2.

### Capturing digital images

For each wine sample, digital images were captured at three different liquid depths (2, 3 and 4 mm) in a 6‐cm diameter polystyrene petri dish. Statistical analyses were conducted on the image‐derived data at each sampling depth to identify the optimal sample volume and height for precise estimation of anthocyanins. To mitigate noise and account for intra‐sample variations, the automated algorithm was programmed to capture 10 consecutive frames for each sample‐depth combination. This multi‐frame approach ensured that the machine learning models could generalize from a broader distribution of pixel data, effectively capturing the nuanced colorimetric profiles of the diverse wine matrices.

### Physicochemical properties of red wine samples

To enhance the predictive capability of regression models, the physicochemical properties of wine samples were also assessed, including alcohol content (v v^−1^) and total soluble solids (°Bx). The volumetric alcohol content of wine samples was measured using a handheld refractometer (AdolfR, Shenzhen, China) which was calibrated with standard ethanol solutions of known concentrations, and the °Bx value was measured using a digital refractometer (ATAGO, Tokyo, Japan).

### TMA concentration

The TMA concentration of wine samples was measured using the pH differential method.^16^ The absorbance of 10‐fold diluted red wines was measured in potassium chloride buffer (pH 1.0) and sodium acetate buffer (pH 4.5) at wavelengths of 510 nm and 700 nm using a UV‐visible spectrophotometer (Lambda XLS & Printer; PerkinElmer, Waltham, MA, USA). The formulas for calculating the TA are:

(1) $$A=\left[A_{510}-A_{700}\right]\mathrm{pH}\left(1.0\right)-\left[A_{510}-A_{700}\right]\mathrm{pH}\left(4.5\right)$$

(2) $$\begin{array}{c} \mathrm{TA}=A\times \mathrm{MW}\times D\times \frac{100}{\varepsilon \times L}\; \end{array}$$

where *A* is the absorbance and *A*_510_ and *A*_700_ stand for absorbance of the sample at the corresponding wavelength. *MW* stands for the molecular weight of malvidin‐3‐glucoside (463.3 g mol^−1^), *D* is dilution factor, *ε* is molar absorptivity (28 000 L g^−1^ mol^−1^) and *L* is the path length of the cuvette (1 cm).

### Statistical analysis

#### Data acquisition

**Figure 3:**
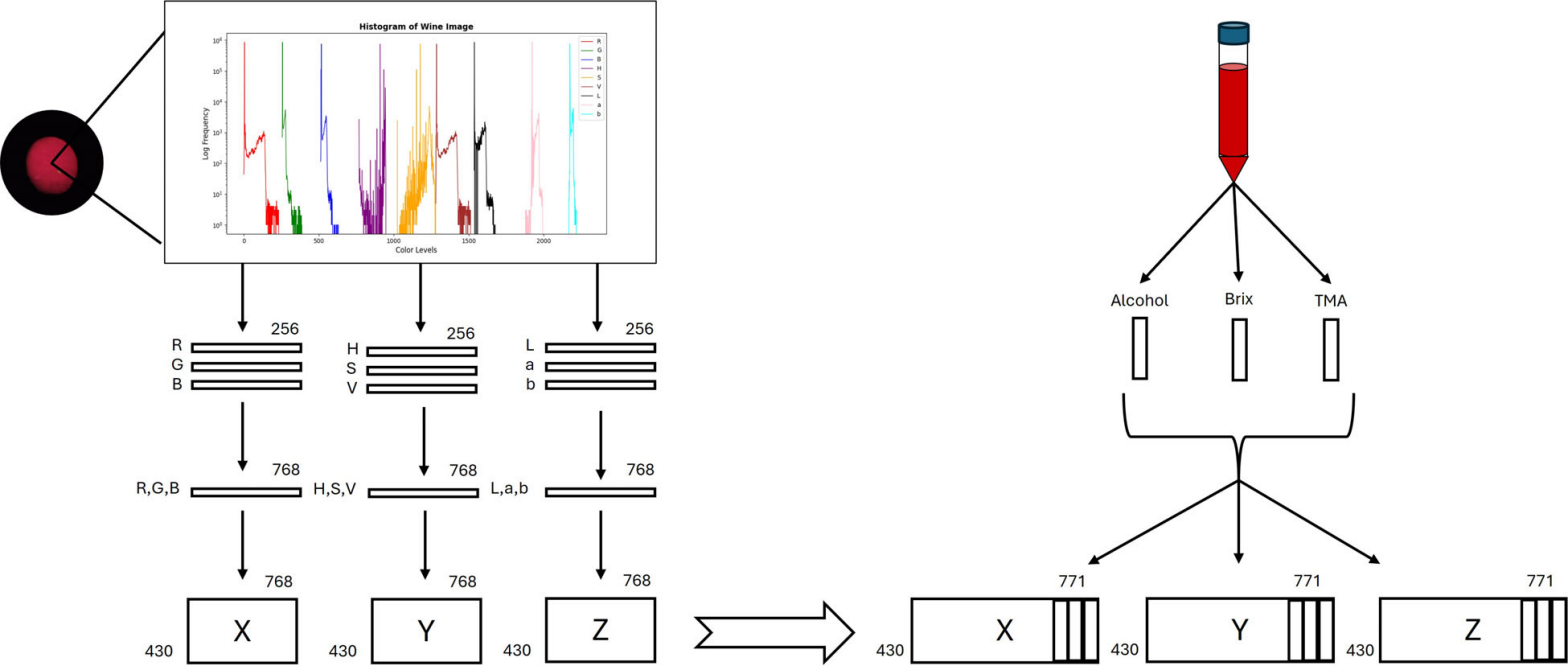
Construction of *X*, *Y*, *Z* matrices.

To predict total monomeric anthocyanin concentration in red wines using data from digital image processing, images of wines were first captured at three liquid depths (2, 3, and 4 mm). For each depth, color features (derived from RGB, HSV and Lab color spaces), physicochemical properties (alcohol content and °Bx) and TMA concentrations were merged into three structured matrices: *X* (RGB), *Y* (HSV) and *Z* (Lab). Each matrix contains 430 rows, corresponding to individual wine images, and 771 columns, representing features (768 color‐derived variables, alcohol, °Bx and TMA). These matrices were organized into separate Excel files (Microsoft Corp., Redmond, WA, USA), with each file containing dedicated sheets for RGB, HSV and Lab data at the respective imaging height. A schematic overview of this workflow is provided in Fig. 3.

#### Data preprocessing

To ensure methodological consistency and reproducibility, all feature matrices were preprocessed using the same workflow for both multivariate and machine learning analyses. First, the dataset was partitioned into training (80%) and testing (20%) subsets for external validation. All preprocessing procedures, including normalization and feature selection, were performed exclusively on the training subset to prevent information leakage into the testing data. Some 80% of data were used for training and 20% were used for testing. The predictors were then normalized. Min–max normalization was applied to color features to prevent dominance by high intensity values. The physicochemical predictors, alcohol and °Bx were normalized using *z*‐score normalization because they have different units and scales. Finally, all TMA values were scaled using log1p transformation to mitigate skewness, which is common in biochemical concentration data, and to stabilize variance.^17^

#### PLSR

The PLSR analysis was performed using Rstudio (Posit PBC, Boston, MA, USA). Random seed was set to 123. A nested cross‐validation framework was used with fiver outer folds for validation and three inner folds for hyperparameter tuning. Nested cross‐validation was employed because it provides a less biased estimate of predictive performance during hyperparameter optimization. The optimal number of latent components (ncomp) was selected via grid search (1–10 components) to minimize root mean square error (RMSE). Final model performance was evaluated on an independent test subset using *R* ^2^ and RMSE. After training, image‐level predictions were aggregated at the sample‐level to account for real sample analysis.

#### RF regression

The RF model was trained using R‐script at random seed 123. The hyperparameters mtry, (number of variables per split) and terminal node size were optimized via grid search within a 5 × 2 repeated cross‐validationframework. Repeated cross‐validation was implemented to reduce variance associated with a single random partitioning of the dataset. Permutation importance was computed to identify key predictors. Final model performance was assessed on the test set using *R* ^2^ and RMSE with sample‐level aggregation to mitigate overfitting.

#### ANN based regression

To predict anthocyanin content, a feedforward neural network (ANN) was implemented using Python TensorFlow at random seed 42. The dataset was split into training (80%) and test (20%) sets using stratified sampling based on anthocyanin quantiles to preserve distribution. Training data were augmented with Gaussian noise (*σ* = 0.02) to improve generalization and robustness against minor imaging variability. This augmentation strategy was applied only to the training subset and was intended to improve model robustness against minor imaging variability. LASSO regression (*α* = 0.001) reduced dimensionality by selecting the 50 most influential features. The ANN architecture included two hidden layers (128 and 32 neurons, rectified linear unit activation) with dropout regularization (30%) and was trained using the Adam optimization algorithm. Early stopping monitored validation loss during training to prevent excessive model fitting. To improve generalization and reduce overfitting risk associated with limited sample size, the framework incorporated multiple regularization strategies, including feature reduction, dropout regularization, early stopping and external validation. Model performance was assessed using *R* ^2^ and RMSE on anthocyanin values. Hyperparameters (learning rate, batch size) were empirically chosen, and reproducibility was ensured through fixed random seeds.

## RESULTS AND DISCUSSION

This study investigated how imaging height and color space influence the performance of three regression models (PLSR, RF and ANN). Images of wines were captured at three liquid depths (2, 3 and 4 mm) and processed in three color spaces (RGB, HSV and Lab, referred to as *X*, *Y* and *Z* feature matrices, respectively). These feature matrices were used to train and test the models for predicting TMA concentration in red wines, and performance was evaluated using *R* ^2^ and RMSE.

### Physicochemical properties of wine samples

The physicochemical properties of the red wine samples are summarized in Table 1.

**Table 1:** TMA concentration and physicochemical properties of red wine samples

Label	TMA (mg L^−1^)	Brix (^°^Bx)	Alcohol (v v^−1^)	Vintage
K1	15.42	7.20	13.64	NA
K2	19.29	8.00	14.77	NA
K3	4.85	8.20	16.29	NA
K4	4.85	7.80	14.77	NA
K5	9.51	6.70	13.26	NA
K6	25.81	8.90	16.29	NA
K7	6.70	10.90	20.83	NA
K8	14.54	7.50	14.02	NA
K9	15.59	6.70	12.50	NA
K10	10.92	8.30	15.53	NA
K11	4.67	8.40	15.91	NA
K12	2.29	6.40	12.50	NA
K13	6.61	8.20	15.53	NA
K14	11.98	8.00	14.77	NA
K15	5.64	11.20	21.59	NA
K16	14.09	7.50	14.02	NA
K17	4.14	6.20	11.74	NA
K18	3.70	8.20	15.53	NA
K19	5.29	6.90	12.50	NA
K20	14.45	6.40	12.12	NA
K21	8.55	8.00	14.77	NA
K22	8.28	11.40	21.59	NA
K23	20.35	8.10	15.15	NA
K24	13.83	7.90	14.77	NA
K25	33.56	7.40	14.02	NA
L23‐42	35.59	9.50	17.05	2023
L23‐36	17.79	9.80	17.80	2023
L23‐28	36.82	7.90	14.77	2023
L23‐19	39.82	10.10	17.80	2023
L23‐54	18.68	8.30	14.77	2023
L23‐3	25.55	8.80	16.29	2023
TK33	60.87	9.20	16.29	2024
TK5	38.85	9.70	17.80	2024
L24‐34	43.16	10.00	18.56	2024
L24‐48	28.81	9.70	17.80	2024
L24‐43	43.08	10.10	17.80	2024
TK32	87.83	8.50	15.53	2024
UK	41.84	8.10	14.77	2024
su1	17.27	8.80	15.53	2023
su2	16.91	8.20	15.53	2023
Fio	5.46	7.80	14.77	2024
Sav	24.67	6.90	12.88	2023
Koroglu	13.21	7.60	14.02	2022

Over time, the total anthocyanin content of red wines changes as a result of various factors. Among these, anthocyanin polymerization and co‐pigmentation play a significant role, leading to a gradual reduction in TMA concentration. For example, Soural *et al*.^18^ have reported a decrease in malvidin 3‐glucoside concentration of red wines during storage from 3 to 30 months. Therefore, the expectation was to observe lower TMA concentration in older wines. As shown in Table 1, most of the wines with vintages of 2024 have higher TMA concentration compared to the vintages of 2023. Moreover, the phenolic composition of wines is largely influenced by the maceration period and the grape variety. It may be inferred that with increasing maceration time, the extraction of phenolic compounds, and consequently anthocyanins, is likely to increase. However, Herjavec *et al*.^19^ have shown that longer maceration times may cause a decrease in the anthocyanin composition of the red wines after a certain threshold. Also, as indicated in Table 1, some younger wines have lower TMA than older wines and the difference between the concentrations may be caused by the differences in the maceration time and grape variety.

### Prediction of TMA using PLSR regression model

For the PLSR model, hyperparameter tuning was performed within each outer fold to select the optimal number of latent components (ncomp). Across all five outer folds, the tuning process consistently selected ncomp = 10, indicating that the full range of components offered the best predictive performance within the cross‐validation framework. Lower latent components (4‐6) resulted in poor *R* ^2^ values compared to the tuned version.

According to Table 2, the PLSR models demonstrated generally strong predictive performance across all conditions with test *R* ^2^ values between 0.772 and 0.944 with RMSE values in the range 0.184–0.371. The highest test *R* ^2^ was observed at a depth of 2 mm in *Y* color space, whereas the lowest *R* ^2^ was observed at a depth of 3 mm in *Y* color space suggesting some sensitivity of model performance to both liquid depth for image acquisition and color space. Overall, color space *Z* consistently produced strong test *R* ^2^ values above 0.91 for all heights compared to other factors.

**Table 2:** Performance metrics of PLSR model

Liquid depth (mm)	Feature matrix	Train *R* ^2^	Test *R* ^2^	Train RMSE	Test RMSE
2	*X*	0.929	0.857	0.210	0.291
	*Y*	0.961	0.944	0.153	0.184
	*Z*	0.948	0.918	0.177	0.225
3	*X*	0.930	0.898	0.207	0.254
	*Y*	0.930	0.772	0.207	0.371
	*Z*	0.932	0.917	0.203	0.225
4	*X*	0.937	0.895	0.196	0.250
	*Y*	0.952	0.907	0.169	0.237
	*Z*	0.953	0.924	0.172	0.215

Although the performance metrics of the models indicate a strong predictive ability, the high number of latent variables raises concerns about practical generalization. For example, Menozzi *et al*.^20^ successfully predicted TMA concentrations from RGB colors using PLSR models with only two latent variables, achieving *R* ^2^ values ranging from 0.81 to 0.85. Although such models offer better generalization, in the context of analytical devices such as the one developed in this study, predictive performance near the lower acceptance threshold may justify maintaining higher model complexity than is typical in standard approaches.

Moreover, Miramont *et al*.^21^ used FTIR spectral data and combined it with PLSR to monitor anthocyanin concentrations of red wines during fermentation. The use of spectral data in combination with PLSR is among the most common prediction methods used in wine quality control and their models achieved test *R* ^2^ values between 0.83 and 0.91 confirming spectral signatures of anthocyanins were a very effective tool for generating predictive models. The performance of the present image‐based PLSR models (test *R* ^2^ up to 0.944) suggests that low‐cost imaging approaches can achieve predictive accuracy comparable to, or even exceeding, that of spectroscopic methods.

To assess whether the differences in PLSR model performance across liquid depths during image acquisition and among color spaces were statistically significant, a two‐way analysis of variance (ANOVA) was performed on the PLSR test *R* ^2^ values. The results indicated that neither liquid depth (*P* > 0.05) nor color space (*P* > 0.05) had a significant impact on the predictive performance of the model. This suggests that, although certain combinations yielded numerically better results, these differences could be the result of random variation.

**Figure 4:**
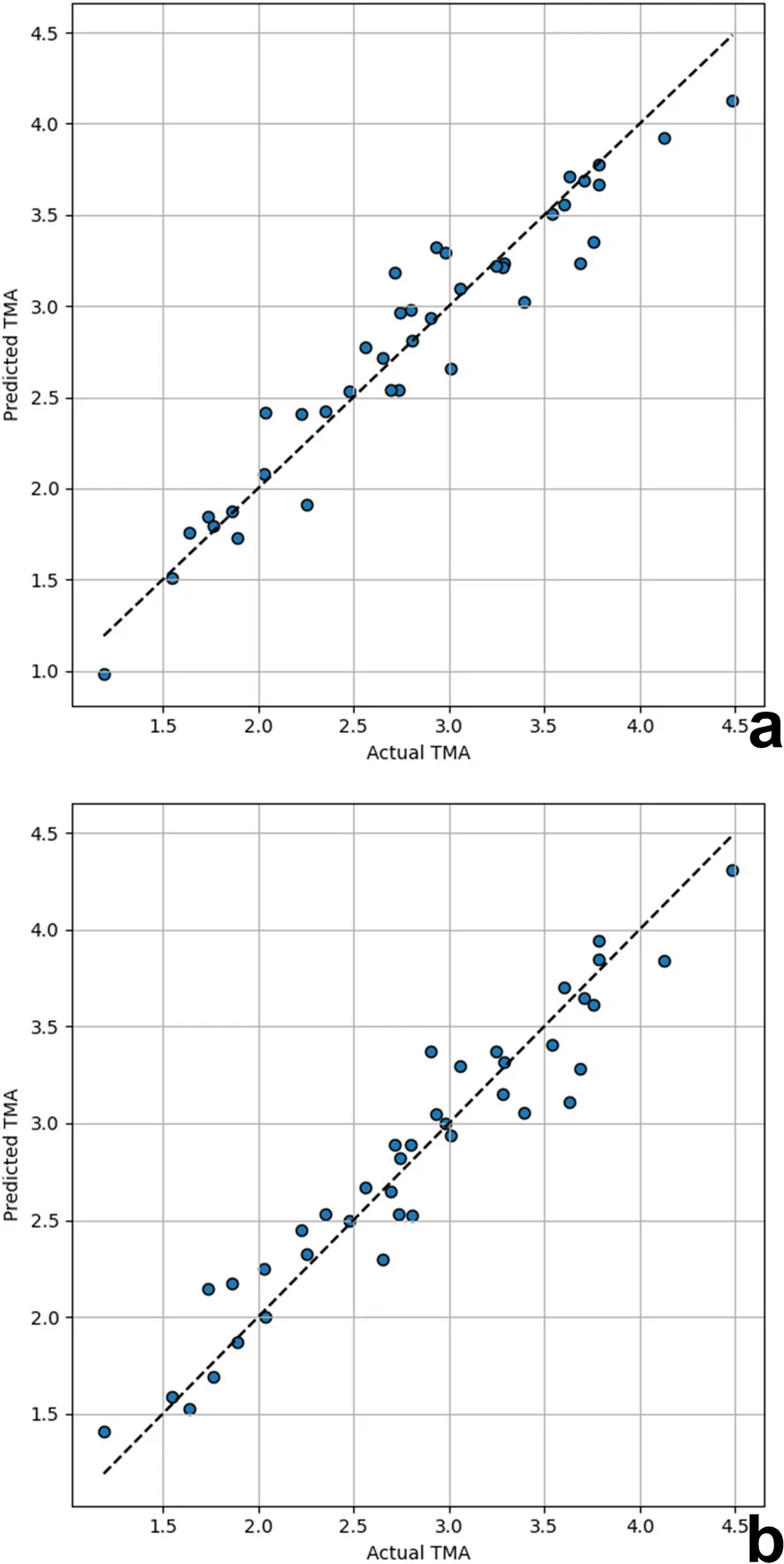
Predicted *versus* actual TMA concentrations for PLSR models at (a) 2‐mm imaging height with HSV features and (b) 4‐mm imaging height with Lab features.

Although the ANOVA results indicated no statistically significant factors, practitioners may still prefer combinations such as 2 mm–*Y* or 4 mm–*Z* for their slightly superior metrics. As shown in Fig. 4, predictions followed the reference line reasonably well across imaging setups, although some dispersion was observed around the mid‐range values. Moreover, the findings imply that the PLSR model is robust across moderate changes in imaging setup and feature representation.

Taken together, the results suggest that the PLSR approach is stable under moderate changes in imaging configuration and feature representation, but its ability to generalize is constrained by the reliance on a relatively high number of latent variables.

### Predictions of TMA using RF regression model

For the RF model, a grid search was conducted across multiple values of mtry (number of predictors randomly sampled at each split: 5, 10, 20, 30, 40, 50) and terminal node size (minimum number of samples per terminal node: 1, 3, 5), using the ‘ranger’ implementation and permutation‐based variable importance. The tuning process was performed within repeated five‐fold cross‐validation loops to reduce bias in model selection. The optimal mtry was consistently found to be 50 across all outer folds, suggesting that a large number of features contributed positively to predictive accuracy. The terminal node size was most frequently selected as 1, indicating that allowing deeper trees with finer splits yielded better performance.

According to the Table 3, the RF model achieved consistently high predictive accuracy, with test *R* ^2^ values ranging from 0.907 to 0.976. The best result was observed at a liquid depth of 2 mm with the *Z* color space, achieving a test *R* ^2^ of 0.976 and test RMSE of 0.137. This suggests strong generalization performance and a good fit between predicted and actual values. Interestingly, although the *Y* color space at a depth of 2 mm also achieved high test *R* ^2^ (0.974), it resulted in the highest test RMSE (0.515) among all configurations, suggesting increased prediction error dispersion, possibly because of sensitivity to specific image characteristics in that representation.

**Table 3:** Performance metrics of RF models

Liquid depth (mm)	Feature matrix	Train *R* ^2^	Test *R* ^2^	Train RMSE	Test RMSE
2	*X*	0.996	0.968	0.062	0.157
	*Y*	0.997	0.974	0.057	0.515
	*Z*	0.997	0.976	0.050	0.137
3	*X*	0.994	0.950	0.075	0.187
	*Y*	0.995	0.907	0.073	0.251
	*Z*	0.995	0.967	0.064	0.153
4	*X*	0.996	0.961	0.058	0.159
	*Y*	0.995	0.952	0.064	0.175
	*Z*	0.994	0.958	0.070	0.169

In a study conducted by Bhardwaj *et al*.^22^ XGBoost regression achieved an *R* ^2^ of 0.94 for predicting total anthocyanins of red wine samples from fluorescence spectra. Both RF and XGBoost are methods based on decision tree architectures, which makes them methodologically comparable, although differing in how trees are constructed and combined to optimize prediction performance. The RF regression model on image‐derived color features achieved a comparable *R* ^2^ of 0.976, highlighting the potential of low‐cost, image‐based approaches to achieve accuracy on par with spectroscopic methods combined with advanced boosting algorithms. However, the difference in performance is strongly related to variations in sample sets, data preprocessing, and hyperparameter tuning procedures.

To test whether these observed performance differences were statistically significant, a two‐way ANOVA was conducted using the test *R* ^2^ scores. The analysis revealed that neither imaging height (*P* > 0.05) nor color space (*P* > 0.05) had a statistically significant effect on model performance. These findings suggest that the RF model is highly stable and not significantly influenced by moderate changes in imaging setup or color features.

**Figure 5:**
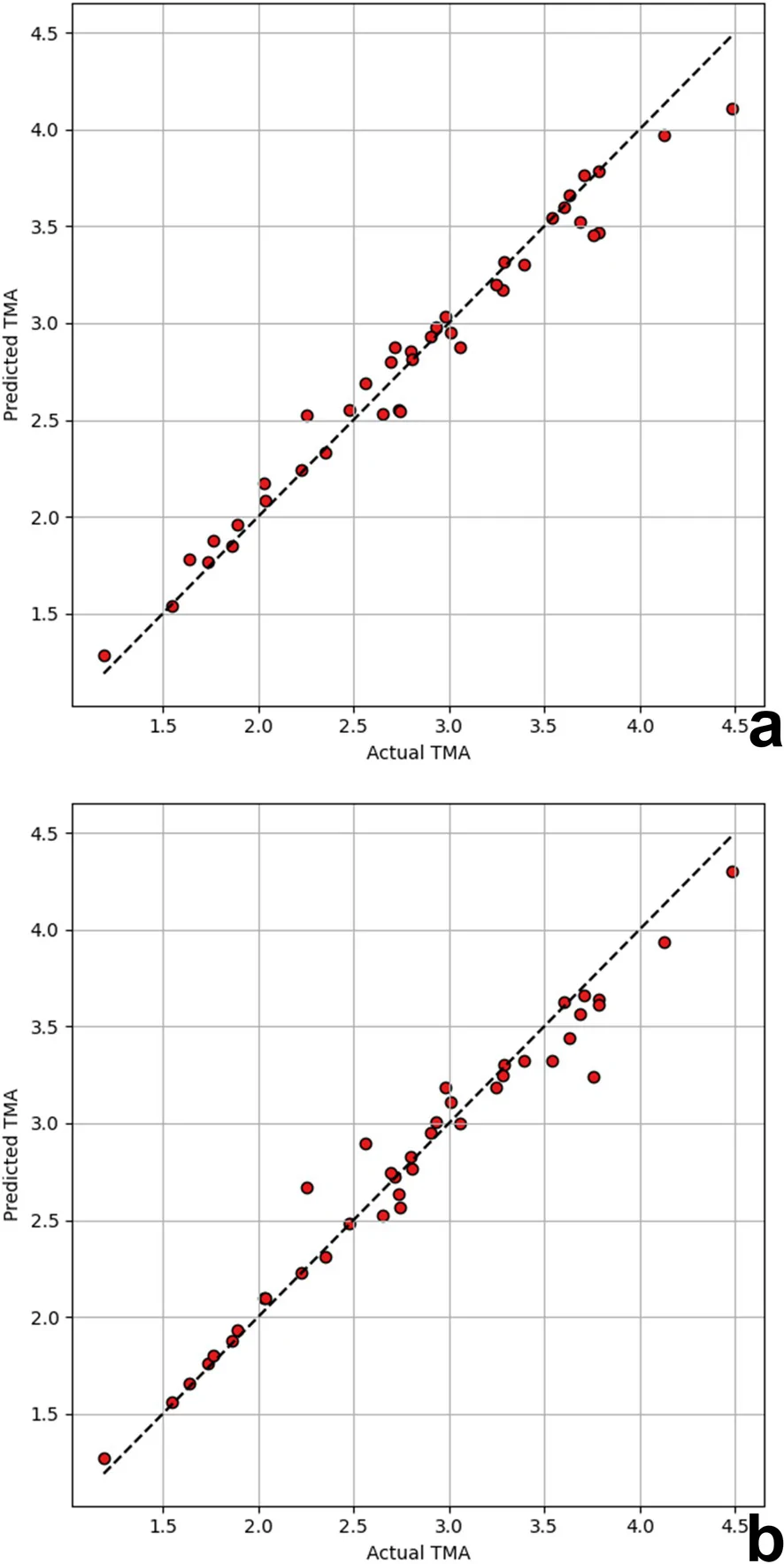
Predicted *versus* actual TMA concentrations for RF models at (a) 2‐mm imaging height with Lab features and (b) 3‐mm imaging height with Lab features.

Figure 5 illustrates the relationship between predicted and actual TMA values under two imaging height conditions. In both cases, the data points are closely aligned with the reference line, reflecting the strong predictive ability of the RF model. Minor deviations were observed in the mid‐range values (2.5–3.0), yet the regression slopes remained close to unity, with test *R* ^2^ values of 0.976 and 0.967 and corresponding RMSE values of 0.137 and 0.153. These results indicate that liquid depth exerts minimal influence on model accuracy.

Overall, these outcomes suggest that the Random Forest model remains highly stable across imaging conditions and is largely unaffected by liquid depth, supporting its applicability in practical, high‐throughput wine analysis.

### Prediction of TMA using ANN‐based regression model

The ANN‐based regression model exhibited high predictive performance under controlled experimental conditions across almost all combinations. The performance metrics of ANN‐based models are presented in Table 4. Notably, the highest test *R* ^2^ of 0.994 and the lowest test RMSE of 0.059 were obtained at a liquid depth of 2 mm for image acquisition using matrix *Z*, suggesting this configuration allowed the model to most effectively capture relevant color information. Matrix *Y* also showed consistently strong performance, particularly at depths of 2 mm and 3 mm, with test *R* ^2^ values of 0.988 and 0.978 respectively. A slight decline in test performance was observed for some combinations at depths of 3 mm and 4 mm, especially with matrix X, indicating that both imaging height and color representation can influence prediction accuracy, even in a robust model such as ANN.

**Table 4:** Performance metrics of ANN models

Imaging height	Feature matrix	Train *R* ^2^	Test *R* ^2^	Train RMSE	Test RMSE
2 mm	*X*	0.991	0.975	0.074	0.121
	*Y*	0.995	0.988	0.057	0.082
	*Z*	0.998	0.994	0.034	0.059
3 mm	*X*	0.989	0.936	0.081	0.193
	*Y*	0.992	0.978	0.068	0.113
	*Z*	0.995	0.984	0.053	0.096
4 mm	*X*	0.996	0.941	0.050	0.185
	*Y*	0.995	0.984	0.053	0.095
	*Z*	0.992	0.975	0.070	0.120

In the study by Taghadomi‐Saberi *et al*.,^23^ ANN models were employed to predict anthocyanin content in sweet cherry using feature engineered parameters derived from digital images. Their best‐performing ANN, with two hidden layers (11–6–20–1 architecture), achieved a correlation coefficient of *r* = 0.98 and an RMSE of 0.67. In comparison, the ANN developed in the present study, trained on raw image features reduced by LASSO regression, attained a test performance of *R* ^2^ = 0.994 with an RMSE of 0.059. This suggests that raw data, when coupled with modern ANN architectures and training strategies, can yield higher predictive power than models based on manually engineered features. One potential explanation could be that use of a deeper neural network with more neurons, dropout regularization, and a modern optimization algorithm (Adam). Although feature‐engineered models may offer greater robustness across varying conditions, the results indicate that deep ANN frameworks are capable of extracting the relevant interactions directly from raw image data, reducing the need for manual preprocessing. Similar applications of ANN with spectral inputs have also been reported, such as in the study by Janik *et al*.,^24^ who predicted total anthocyanins in grape homogenates from visible‐near infrared spectra using ANN models. Together, these findings support the potential of ANN as a powerful predictive framework across different modalities, with the present study extending its application to digital image‐derived color features in wine.

The two‐way ANOVA assessed whether variations in liquid depth and feature matrix significantly impacted test *R* ^2^ values. The effect of liquid depth was not statistically significant (*P* > 0.05), suggesting performance remained stable across depths. However, the type of feature matrix had a statistically significant effect (*P* < 0.05), meaning that the choice of color representation (e.g. *X*, *Y* or *Z*) plays a critical role in model performance.

**Figure 6:**
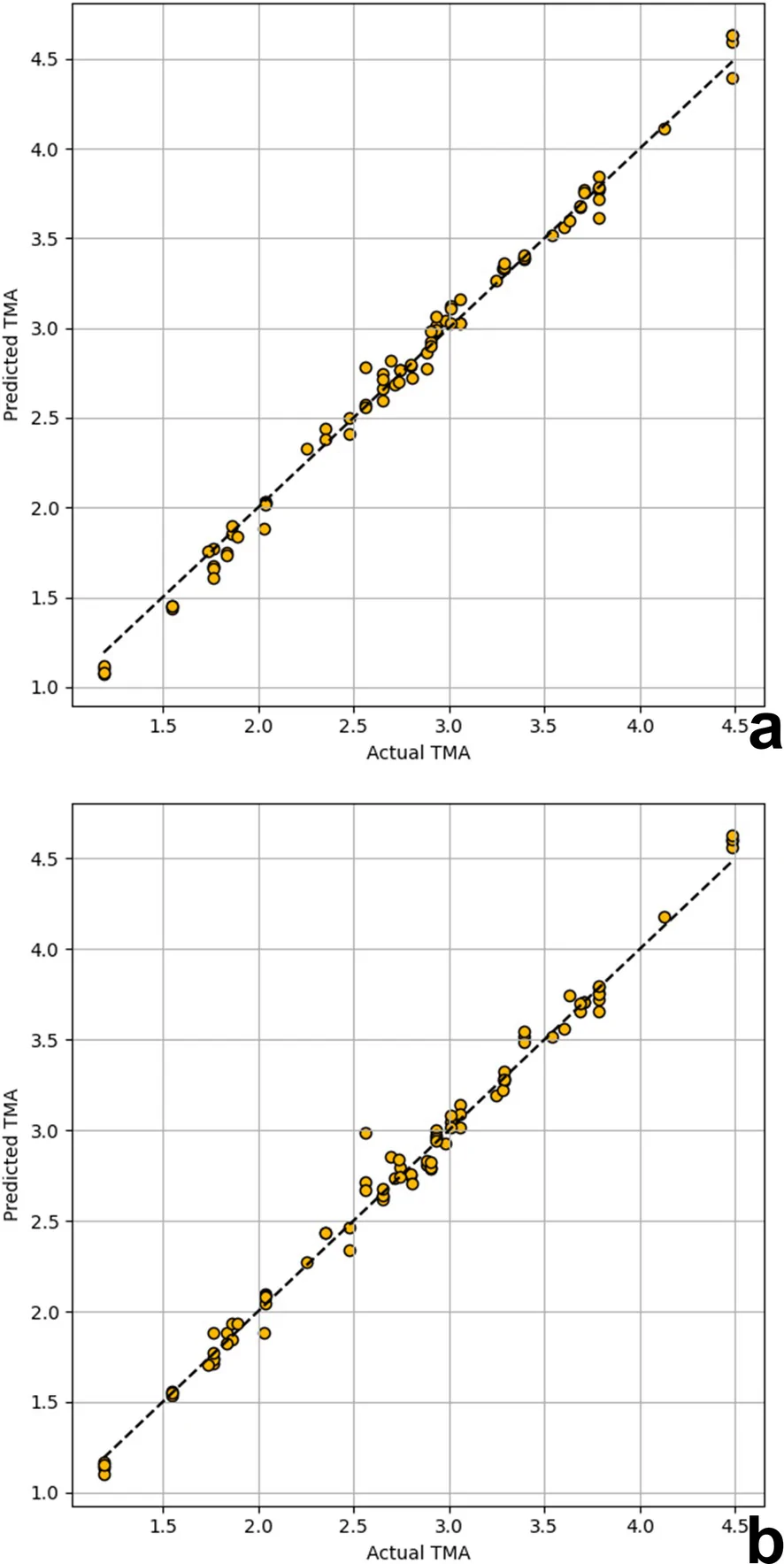
Predicted *versus* actual TMA concentrations for ANN‐based models at (a) 2‐mm imaging height with Lab features and (b) 2‐mm imaging height with HSV features.

As shown in Fig. 6, the ANN models effectively captured the non‐linear relationship between colorimetric features and TMA, yielding predictions closely aligned with the experimental values. Unlike the RF models, where performance was largely unaffected by imaging conditions, the ANN results demonstrated clear sensitivity to the type of feature matrix. Dispersion around the regression line was markedly lower when matrix *Z* was applied, consistent with the higher *R* ^2^ and lower RMSE values obtained under this configuration.

Taken together, these findings demonstrate that ANN models offer high reliability and generalization across imaging conditions, with performance being primarily shaped by the choice of feature matrix rather than liquid depth. The strongest performance was consistently achieved with Lab‐derived features, highlighting their suitability for non‐linear modeling approaches in digital wine analysis.

### Comparison of model performance

The optimal configuration for each algorithm, PLSR at a liquid depth of 2 mm with HSV features (test *R* ^2^ = 0.944, RMSE = 0.184), RF at 2 mm with Lab features (*R* ^2^ = 0.976, RMSE = 0.137) and ANN at 2 mm with Lab features (*R* ^2^ = 0.994, RMSE = 0.059) was first identified. Optimal digital image acquisition was achieved at a liquid depth of 2 mm, corresponding to a sample volume of 5.6 mL. Moreover, to obtain statistically robust estimates of each model's predictive ability, and to guard against a lucky (or unlucky) single train–test split, a stratified five‐fold cross‐validation on the entire dataset for each algorithm in turn was conducted. Stratification was performed on quintiles of the target variable (TMA) to preserve the original distribution in each fold. Within each outer fold, full pipelines of PLSR, RF and ANN were run. Finally, the test *R* ^2^ and RMSE is then recorded. This procedure yields five independent performance measures per model, which is given in Table 5 with the mean ± SD, giving a more reliable picture of real‐world generalization.

**Table 5:** Mean test *R* ^2^ and RMSE values and standard deviations of five‐fold CV results

Model	Mean test R^2^	Mean test RMSE
PLSR	0.897 ± 0.019^B^	0.252 ± 0.032^A^
RF	0.960 ± 0.011^A^	0.174 ± 0.023^B^
ANN	0.973 ± 0.020^A^	0.113 ± 0.037^B^

*Note:* Mean ± SD values within a column followed by different letters are significantly different (*P* < 0.05).

A Tukey post‐hoc analysis of the five‐fold cross‐validated test *R* ^2^ means assigned RF and ANN models to group A and PLSR to group B. The tests confirmed that PLSR underperformed compared to RF and ANN (*P* < 0.01), whereas the difference between RF and ANN was not significant. Thus, these results demonstrate that non‐linear methods (RF, ANN) substantially outperform linear PLSR models in this imaging context and, although ANN achieves the highest average accuracy, its advantage over RF is not statistically robust, making RF an almost equivalent alternative when training speed or interpretability is a priority.

Despite the strong predictive performance obtained in this study, several limitations should be acknowledged. The dataset consisted of 43 unique wine samples acquired under controlled laboratory conditions, and therefore the reported performance metrics may not fully represent variability encountered in industrial‐scale or cross‐regional wine datasets. Although repeated imaging substantially increased the number of observations available for model training, the chemical diversity of the dataset ultimately remains constrained by the number of unique wines. Furthermore, imaging conditions, illumination geometry, camera characteristics and sample presentation were standardized in this work, meaning additional studies are required to evaluate model transferability across different hardware configurations and real‐world environments.

Nevertheless, the incorporation of external hold‐out testing, stratified cross‐validation, nested validation procedures, and statistical comparison of model performance provides evidence that the developed models captured reproducible relationships between image‐derived color features and anthocyanin concentration under the experimental conditions employed in this study.

## CONCLUSIONS

In the present study, three regression models (PLSR, RF and ANN), were evaluated for predicting total monomeric anthocyanin content from digital image features extracted at three liquid depths for image acquisition and in three color spaces. After identifying each model's optimal configuration on a hold‐out set, stratified five‐fold cross‐validation was conducted to obtain robust performance estimates. A one‐way ANOVA confirmed significant differences among the three models, and Tukey's honestly significant difference grouped RF and ANN models together and PLSR model separately. These results demonstrate that the non‐linear methods (RF and ANN) significantly outperform the linear PLSR model (*P* < 0.05), whereas the accuracy of RF is statistically indistinguishable from ANN (*P* > 0.05). Given its near‐equivalent performance and typically faster training, RF offers an attractive balance of accuracy, interpretability and computational efficiency. However, because red wine is often positioned as a high‐value, premium product, where small differences in anthocyanin content can affect sensory quality and consumer perception, it may be preferable in certain applications to prioritize absolute predictive accuracy over model simplicity. In such cases, the slight but consistent edge of the ANN could justify its additional computational and implementation complexity. Although the present study should be interpreted as a proof‐of‐concept investigation, the findings demonstrate the strong potential of combining low‐cost digital imaging with machine learning for rapid anthocyanin estimation in red wines. To further enhance model generalizability and robustness, future studies should expand the training set to include wines from a broader range of cultivars to introduce variability in anthocyanin profiles and help the models learn more universal feature patterns.

## FUNDING INFORMATION

This work was supported by the Scientific and Technical Research Council of Turkey (TÜBİTAK, Program No: 2209‐A Research Project Support Program for Undergraduate Students).

## CONFLICTS OF INTEREST

The authors declare that they have no conflicts of interest.

## ETHICAL STATEMENT

Ethics approval was not required for this research.

## AUTHOR CONTRIBUTIONS

EA, BB and SU were responsible for conceptualization. EA was responsible for methodology. EA and BB were responsible for formal analysis and investigation. EA was responsible for writing the original draft. BB and SU were responsible for reviewing and editing. BB was responsible for funding acquisition. BB and SU were responsible for supervision.

## Data Availability

The data that support the findings of this study are available from the corresponding author upon reasonable request.
